# SARS-CoV-2 infection prevalence and associated factors among primary healthcare workers in France after the third COVID-19 wave

**DOI:** 10.1038/s41598-024-55477-9

**Published:** 2024-03-05

**Authors:** Marie Pouquet, Dorine Decarreaux, Laura Di Domenico, Chiara E. Sabbatini, Pol Prévot-Monsacre, Toscane Fourié, Paola Mariela Saba Villarroel, Stephane Priet, Hélène Blanché, Jean-Marc Sebaoun, Jean-François Deleuze, Clément Turbelin, Louise Rossignol, Andréas Werner, Fabienne Kochert, Brigitte Grosgogeat, Pascaline Rabiega, Julien Laupie, Nathalie Abraham, Harold Noël, Sylvie van der Werf, Vittoria Colizza, Fabrice Carrat, Remi Charrel, Xavier de Lamballerie, Thierry Blanchon, Alessandra Falchi

**Affiliations:** 1 Sorbonne Université, INSERM, Institut Pierre Louis d’Epidémiologie et de Santé Publique (IPLESP), 75012 Paris, France; 2 Laboratoire de Virologie, Université de Corse Pascal Paoli, UR7310 Bioscope, 20250 Corte, France; 3 Unité Des Virus Emergents, Aix Marseille University, IRD 190, INSERM U1207, 13005 Marseille, France; 4 Fondation Jean Dausset-CEPH, 75010 Paris, France; 5 Association Française de Pédiatrie Ambulatoire (AFPA), Zone de la Fouquetière, 155 Rue Edouard Branly, 44150 Ancenis-Saint-Géréon, France; 6 Faculté d’Odontologie, Université Claude Bernard Lyon 1, Université de Lyon, 69000 Lyon, France; 7 Laboratoire des Multimatériaux et Interfaces, UMR CNRS 5615, Université Claude Bernard Lyon 1, Université de Lyon, 69000 Lyon, France; 8 Réseau ReCOL, Association Dentaire Française, 75000 Paris, France; 9 Service d’Odontologie, Hospices Civils de Lyon, 69007 Lyon, France; 10 IQVIA, Réseau de Pharmaciens, 75000 Paris, France; 11 Infectious Diseases Division, Santé Publique France, 94410 Saint Maurice, France; 12 Institut Pasteur, Université Paris Cité, CNRS UMR3569, Molecular Genetics of RNA Viruses Unit, 75015 Paris, France; 13 Institut Pasteur, Université Paris Cité, National Reference Center for Respiratory Viruses, 75015 Paris, France; 14 Département de Santé Publique, Assistance Publique-Hôpitaux de Paris, Hôpital Saint-Antoine, Sorbonne Université, 75012 Paris, France; 15 LE Service de Prévention du Risque Infectieux (LESPRI), CLIN AP-HM Hôpitaux Universitaires de Marseille, 13005 Marseille, France

**Keywords:** SARS-CoV-2, COVID-19, Healthcare workers, Primary healthcare, General population, Prevalence, Risk factors, Risk factors, Infectious diseases

## Abstract

Data on the SARS-CoV-2 infection among primary health care workers (PHCWs) are scarce but essential to reflect on policy regarding prevention and control measures. We assessed the prevalence of PHCWs who have been infected by SARS-CoV-2 in comparison with modeling from the general population in metropolitan France, and associated factors. A cross-sectional study was conducted among general practitioners (GPs), pediatricians, dental and pharmacy workers in primary care between May and August 2021. Participants volunteered to provide a dried-blood spot for SARS-CoV-2 antibody assessment and completed a questionnaire. The primary outcome was defined as the detection of infection-induced antibodies (anti-nucleocapsid IgG, and for non-vaccinees: anti-Spike IgG and neutralizing antibodies) or previous self-reported infection (positive RT-qPCR or antigenic test, or positive ELISA test before vaccination). Estimates were adjusted using weights for representativeness and compared with prediction from the general population. Poisson regressions were used to quantify associated factors. The analysis included 1612 PHCWs. Weighted prevalences were: 31.7% (95% CI 27.5–36.0) for GPs, 28.7% (95% CI 24.4–33.0) for pediatricians, 25.2% (95% CI 20.6–31.0) for dentists, and 25.5% (95% CI 18.2–34.0) for pharmacists. Estimates were compatible with model predictions for the general population. PHCWs more likely to be infected were: GPs compared to pharmacist assistants (adjusted prevalence ratio [aPR] = 2.26; CI 95% 1.01–5.07), those living in Île-de-France (aPR = 1.53; CI 95% 1.14–2.05), South-East (aPR = 1.57; CI 95% 1.19–2.08), North-East (aPR = 1.81; CI 95% 1.38–2.37), and those having an unprotected contact with a COVID-19 case within the household (aPR = 1.48; CI 95% 1.22–1.80). Occupational factors were not associated with infection. In conclusion, the risk of SARS-CoV-2 exposure for PHCWs was more likely to have occurred in the community rather than at their workplace.

## Introduction

Healthcare workers (HCWs) play a crucial role as frontline responders during infectious disease outbreaks such as the coronavirus disease 2019 (COVID-19). Protecting them from infection is vital to ensure their own health, to maintain continuous patient care and to prevent HCW-to-patient contamination^1^. The World Health Organization estimated that between 80,000 and 180,000 HCWs could have died from the COVID-19 between January 2020 and May 2021^1^. Throughout the pandemic, the prioritized protection of HCWs has relied on the use of personal protective equipment (PPE), the implementation of preventive measures in the workplace, and the rapid access to vaccination^1,2^. Measuring the extent of the SARS-CoV-2 infection among HCWs is essential to reflect on policy regarding prevention and control measures.

Previous studies showed substantial variability in prevalence and risk factors for SARS-CoV-2 infection among HCWs, attributed to different job roles, exposure to COVID-19 patient, access to PPE, data collection periods, and community prevalence^3,4^. Some studies reported a higher risk (between two- and seven-fold) of infection among HCWs from hospital or other healthcare settings than in the general population^5^, while others have not found such differences^4,6,7^. While some authors reported a dose–response association between COVID-19-patient exposure and the risk of SARS-CoV-2 infection^8,9^, others showed that community exposure was associated with infection but workplace factors were not^10–12^. Several studies have also suggested that HCWs in primary care were at higher risk of infection than in hospital settings due to lower availability of PPE, in addition to a high flow of patients^13,14^. However, most studies published to date were conducted among hospital HCWs. In France, the extent of the SARS-CoV-2 infection among general practitioners (GPs), pediatricians and pharmacist workers in primary care remains unknown despite their significant involvement throughout the pandemic, particularly in carrying out COVID-19 tests and vaccination. Some PHCWs, such as dentists, have also extended their support beyond their usual practices^15^. Providing data of SARS-CoV-2 infection in these populations is crucial in a context of calls to better integrate them into the planning of health emergency responses^16^.

Our aims were to (1) assess the prevalence of PHCWs infected with SARS-CoV-2 infection in metropolitan France after the third COVID-19 wave among several populations, including GPs, pediatricians, dentists and pharmacy workers; (2) compare these estimates with those obtained through mathematical modeling for the general population; (3) identify associated factors.

## Methods

### Study design and PHCWs recruitment

We used data from the COVID-SéroPRIM study described elsewhere^17,18^. Briefly, this nationwide cross-sectional study was conducted between May 10, 2021 and August 31, 2021 among GPs, pediatricians, dental workers (dentists and assistants), and pharmacy workers (pharmacists and assistants) in primary care thorough metropolitan France. The survey was conducted after the third wave of COVID-19 in France. COVID-19 vaccination of HCWs was available for HCWs without limitations from early February 2021.

The PHCWs were recruited from the following four primary care research and monitoring networks: the French Sentinelles Network (GPs), which collects real-time epidemiological data from 1338 GPs (2.3% of French GPs) for surveillance and research purposes^19^, the French Association of Ambulatory Pediatrics, a nonprofit association with 1500 pediatricians (71.8% of French pediatricians) that aims to promote medical research in the field of ambulatory pediatrics, the Clinical Research in Liberal Dentistry (ReCOL) network, a national research network with 830 liberal dentists (2.4% of French dentists), and IQVIA (pharmacy workers), an international company that collects data from 14,000 records of retail pharmacies (50.2% of French pharmacies). This recruitment process based on pre-existing networks allowed us to rapidly initiate and collect data, which was crucial at that time of the pandemic, and to ensure that the study was properly designed according to professional specialty. The PHCWs were invited by each network to enroll in the study via e-mail communication, virtual meetings, and announcements on the social media platforms for each network. Volunteers could register for the study by following a link to the COVID-SéroPRIM study website. All PHWCs were eligible to participate in the study except those who had previously taken part in a clinical trial for chemoprophylaxis against SARS-CoV-2 infection. Electronic informed consent was obtained from each participant before their enrolment in the study.

### Data collection and serological analysis

After providing online consent, PHCWs were invited to fill a self-administered electronic questionnaire and to perform a capillary blood sampling. Participants had access to the online questionnaire on the COVID-SéroPRIM study website, where they could log in using their own identifier. The questionnaire collected data on socio-demographic characteristics, household size and composition, smoking status, clinical characteristics (chronic disease, history of SARS-CoV-2 RT-PCR/antigenic or ELISA testing, and of COVID-19 vaccination), history of unprotected COVID-19 case contact (defined as face-to-face contact with a confirmed COVID-19 case without the use of recommended PPE), and occupational activities during the first lockdown and the following period (place of work, care of COVID-19 patients, performance of COVID-19 tests, use of PPE).

PHCWs received a dried-blood collection card (DBS) kit to be returned to the centralized biobank (CEPH Biobank, Paris, France) after self-sampling of capillary blood. Samples were prepared and send for serological analyses (Unité des Virus Emergents, Marseille, France). More details on serological methods can be found in previous published work^17,18^.

All samples were tested for IgG antibodies against the Spike (S) and the Nucleocapsid (N) proteins as well as neutralizing activity against SARS-CoV-2. An ELISA test (Euroimmun^®^, Lübeck, Germany) was used to detect anti-SARS-CoV-2 IgG against the S1 domain of the S protein (ELISA-S). In accordance with the manufacturer’s instructions, a test was considered ELISA-S-positive if the sample density ratio ≥ 1.1 (sensitivity, 87%; specificity, 97.5%)^20^. An immunoassay on Luminex was used to detect IgG directed against the N-protein (CTD and NTD domains, N-immunoassay). The cut-off values and assay performance indicators were calculated by receiver operating characteristic curve (ROC) analysis^21^. The specificity/sensitivity values for the CTD and NTD domains in the duplex assay were 96.1%/97.8% and 87.8%/88.5%, respectively. An in-house microneutralization assay was used to detect neutralizing anti-SARS-CoV-2 antibodies^22^. The neutralization titer referred to the highest dilution of serum with a positive result. Specimens with a VNT titer ≥ 20 were considered positive.

### Outcome

The main outcome was a history of SARS-CoV-2 infection, as defined by the following criteria. (1) A positive N-immunoassay from DBS samples. Indeed, anti-N antibodies are not elicited by COVID-19 vaccines that target the S protein, including all vaccines that had been used in France at the time of the survey, and are developed as a result of SARS-CoV-2 infection^23^. However, using anti-N antibodies alone as a marker for natural infection may be problematic as anti-N antibodies have been shown to wane quickly in the first months after infection^24^. Thus, to avoid the risk of misclassification among individuals with negative N-immunoassay despite previous infection, SARS-CoV-2 infection was also determined by the following information: (2) a positive S-ELISA or neutralizing assay from DBS samples in unvaccinated individuals; (3) a self-reporting of a positive SARS-CoV-2 confirmed by reverse transcription-quantitative polymerase chain reaction (RT-qPCR) or antigenic test; (4) a self-reporting of a positive ELISA test (before the first dose in vaccinated individuals) (See Supplementary Table S1 for details).

### Covariables

PHCWs were categorized in 6-level group categories according to occupation. Non-occupational factors potentially associated with SARS-CoV-2 infection comprised: age (< 40/40–49/50–59/> 60), sex, household factors (number of adults: 1/2/≥ 3; of children: 0/≥ 1; of rooms: < 3/3/≥ 4), comorbidities (obesity, hypertension, diabetes, others chronic diseases), smoking status, unprotected contact with a COVID-19 case and region of workplace (a five-category variable defined according to the telephone area code and consistent with the various degrees of pandemic intensity across the regions: Île-de-France/North-West/North-East/South-East/South-West). Occupational factors included place of work (primary care only/other place), number of days worked per week (< 3/3–4/> 4), care of COVID-19 patients, performance of COVID-19 test, occupational activities during the first lockdown and access to PPE (FFP2 or surgical mask, gloves and coat, glasses and coverall).

### Statistical analysis

Categorical variables were described by numbers and percentages, with comparison using Chi-square test or Fisher’s exact test when appropriate.

Region- and age-weighted prevalences were estimated for GPs, pediatricians, dentists and pharmacists. Our weights were the age-region proportion in the population (from the French 2021 census for each population) divided by the age-region proportion in our sample, for each age-region combination (Supplementary Table S2). Since national data were not available for dental and pharmacist assistants, we could not estimate weighted prevalence for these groups. 95% confidence intervals (CI) for estimates were Wald-type intervals computed on the log-odds scale, as implemented in the R “survey” package.

We compared the estimates among PHCWs with the proportion of the general population that has been infected by the SARS-CoV-2, at the national and regional levels, obtained with a mathematical model fitted to the virus spread in France^25^. For the national estimate, we used a stochastic age-stratified transmission model, integrating data on demography, age profile, and social contacts for the French population^25^. Four age classes were considered: [0–11), [11–19), [19–65) and 65+ years old. Transmission dynamics follows a compartmental scheme where individuals are divided into susceptible, exposed, infectious, hospitalized and recovered. For the regional estimates, we used a stochastic metapopulation transmission model, with individuals divided in the 12 regions of mainland France (excluding Corsica). Regions are interconnected by coupling probabilities, inferred from mobility data. Both models were parameterized with estimates from the literature on the infection-hospitalization ratio, and were fitted to hospital admission data, to reproduce the observed epidemic and estimate the total number of infections. Model predictions were validated against serological estimates [Pullano, 2021 #268]. We extracted the predicted proportion of SARS-CoV-2 infection as of June 1, 2021. Median and 95% probability ranges of the estimated proportions were computed from 100 independent stochastic runs for each model. We compared the estimates for adults (age bracket [19–65)) at national level with the prevalences among PHCW (non-overlapped 95% CI and probability ranges indicating a statistical difference between the two estimates). We analyzed the correlation of the estimates among PHCWs and the general population by using Pearson correlation test.

Poisson regression models with robust standard errors were used on unweighted data to identify the factors associated with the SARS-CoV-2 infection. A backward elimination procedure was used. The initial multivariable model included all factors with a p-value < 0.20 in the univariable models. Elimination of covariates was based on the significance of the Wald chi-square test for parameter estimates (p-value < 0.05). Improvement of model fit was determined through the Akaike Information Criterion (lowest value was preferable). To account for possible interactions, we compared model fit before and after addition of an interaction term between occupational group and occupational exposure factors using likelihood ratio tests, with interactions included where p-value < 0.05 for the likelihood ratio test. Missing value were excluded from analysis. Statistically significant was considered if p value ≤ 0.05. All the analyses were computed with R software, version 4.0.3 (4.0.3, R Core Team, 2021, R Foundation for Statistical Computing, Vienna, Austria; https://www.r-project.org/), with the R packages “ggplot2”, “dplyr”, "sandwich".

### Ethical statements

The COVID-SéroPRIM study was approved by the ethical committee Île de France V (Paris, France, registration number ID RCB: 2020-A03298-31). All research was performed in accordance with relevant guidelines and regulations for research involving human beings, with all methods conducted in accordance with the Declaration of Helsinki. Electronic informed consents were obtained from each participant before enrolment.

### Ethics approval and consent to participate

This study has been approved by the ethics committee “CPP Île de France V” (ID RCB: 2020-A03298-31) and the National Data Protection Agency (CNIL, registration number MLD/MFI/AR213778). The protocol described in this article is V.3 of the COVID-SéroPRIM study protocol approved on March 5 2020. Inserm is the sponsor of this study. Any substantial amendment to the protocol will be submitted to the sponsor and sent to the ethics committee for approval before implementation. Informed consent was obtained from all subjects involved in the study.

## Results

### Participants

**Figure 1:**
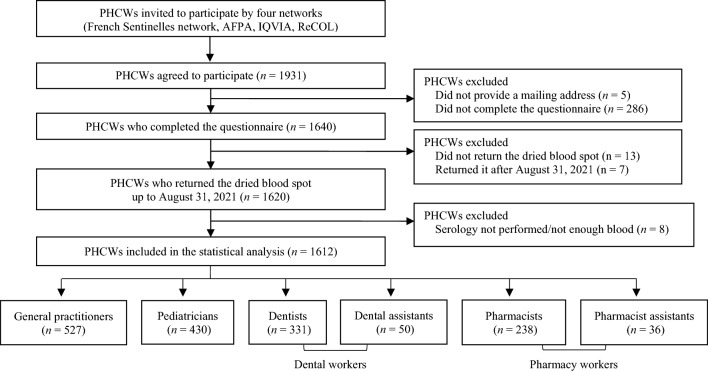
Flow chart of primary healthcare worker (PHCW) participants, COVID-SéroPRIM study, France, May–August 2021.

The recruitment of the study participants has been previously described and details are showed in flow chart in Fig. 1^18^.

A total of 1612 PHCWs were included in the analysis, including 32.7% GPs (n = 527; participation rate: 39.4%; coverage: 0.9% of the French GPs), 26.7% pediatricians (n = 430; participation rate: 28.7%; coverage: 20.6% of the French pediatricians), 20.5% dentists (n = 331; participation rate: 39.9%; coverage: 1.0% of the French dentists), 3.1% (n = 50) dental assistants, 14.8% pharmacists (n = 238; participation rate: 1.7%; coverage: 0.9% of the French pharmacy) and 2.2% (n = 36) pharmacist assistants.

### Non-occupational and occupational factors

PHCW characteristics for the overall population and for each occupational group are showed in Supplementary Table S3. Briefly, the median age of PHCWs was 47 years (Interquartile Range 39–57), and 69.0% (n = 1112) were women. There were 64.5% (n = 1039) living with one adult (≥ 18 year-old), 24.1% (n = 388) with two adults and 44.8% (n = 588) with at least one child (< 18 year-old). Of PHCWs, 23.3% (n = 376) had an unprotected contact with a COVID-19 case, most frequently within household (11.0% of all PHCWs; n = 178), or with colleagues (6.3%; n = 101).

Regarding their occupational characteristics, 85.3% (n = 1375) were working exclusively in primary care settings, 71.3% (n = 1149) were involved in care of COVID-19 patients (ranging from 12.0 to 97.5% according to occupational groups, p < 0.0001), and 30.4% (n = 483) performed COVID-19 test (from 2.0 to 48.5% according to occupational groups, p < 0.0001). During the first lockdown, 5.0% (n = 80) worked in face-to-face without using FFP2 or surgical mask every day (from 2.0 to 11.1% according to occupational groups, p < 0.0005). All occupational characteristics were statistically different according to occupational groups (*p* < 0.001) (see Supplementary Table S3 for further details).

### Unweighted and weighted prevalences

A total of 457 PHCWs (28.3%) had a history of SARS-CoV-2 infection. Supplementary Tables S4–S7 report more details about SARS-CoV-2 infection among PHCWs. Among the 457 PHCWs with a history of SARS-CoV-2 infection, 381 (83.4%) had anti-N antibodies. Of the remaining 75 (16.6%) classified as having a history of SARS-CoV-2 infection, 13 (2.8%) non-vaccinees had anti-S antibodies, 45 (9.9%) had a history of a positive RT-qPCR or antigenic test, and 18 (3.9%) had a history of a positive ELISA test before the vaccination (Table S1).

Overall, among the 1345 (83.4%) PHCWs having concordant results between the serology and the self-reported history of SARS-CoV-2 infection, 1155 (71.6%) had a positive serology and a self-reported history of infection, and 190 (11.8%) had a negative serology and no self-reported history of infection (Table S4). A total of 267 (16.6%) PHCWs had discordant results: 204 (12.7%) had a positive serology without any report of infection, and 63 (3.9%) had a negative serology with a self-reported history of infection.

Unweighted and weighted prevalence of PHCWs that have been infected by SARS-CoV-2 infection are presented in Table 1. Region- and age-weighted prevalences by occupation were: 31.7% (95% CI 27.5–36.0) for GPs, 28.7% (95% CI 24.4–33.0) for pediatricians, 25.2% (95% CI 20.6–31.0) for dentists, and 25.5% (95% CI 18.2–34.0) for pharmacists.

**Table 1:** Unweighted and region-age weighted prevalence estimates for previous or current SARS-CoV-2 infection among PHCWs and by occupational group in France, COVID-SéroPRIM study, May–August 2021.

SARS-CoV-2 infection	GPs n = 527	Pediatricians n = 430	Dentists n = 331	Dental assistants n = 50	Pharmacists n = 238	Pharmacist assistants n = 36	Total n = 1612
Number	169	120	93	11	59	5	457
Prevalence (confidence interval)							
Unweighted	32.1 (28.1, 36.1)	27.9 (23.7, 32.1)	28.1 (23.3, 32.9)	22.0 (10.5–33.5)	24.8 (19.3, 30.3)	13.9 (2.6, 25.2)	28.3 (26.1, 30.6)
Region-age weighted	31.7 (27.5, 36.0)	28.7 (24.4, 33.0)	25.2 (20.6, 31.0)	n/a	25.5 (18.2, 34.0)	n/a	

n/a: non-applicable, since no data were available about the distribution of dental assistants and pharmacist assistants in metropolitan France to estimate region-age weighted prevalence for these two populations.

### Prevalences among PHCWs in comparison with modelling from the general population

**Figure 2:**
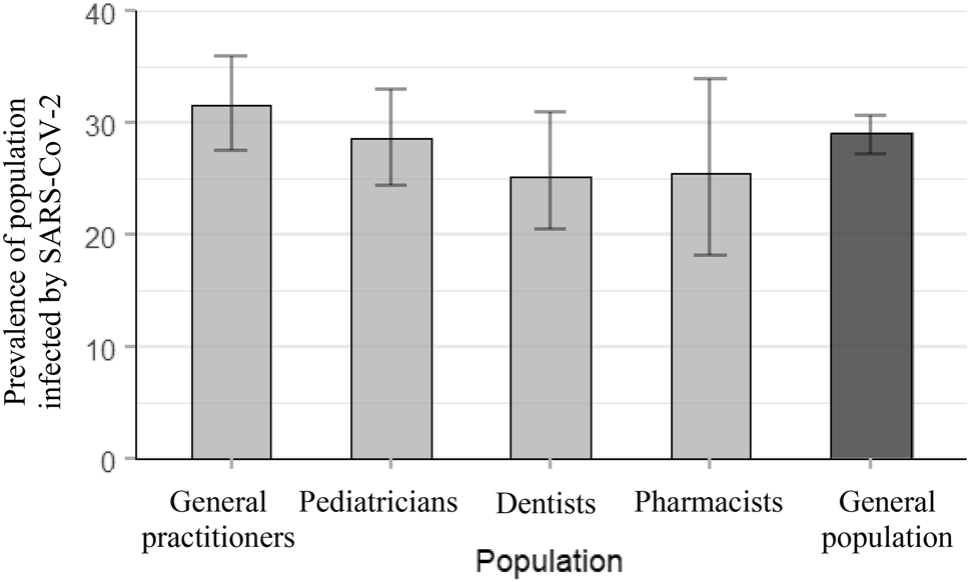
Estimates of PHCWs (COVID-SéroPRIM study, France, May–August 2021) and of the general adult population (from mathematical modeling) that have been infected by the SARS-CoV-2 in metropolitan France after the third COVID-19 wave (as of June 1, 2021).

**Figure 3:**
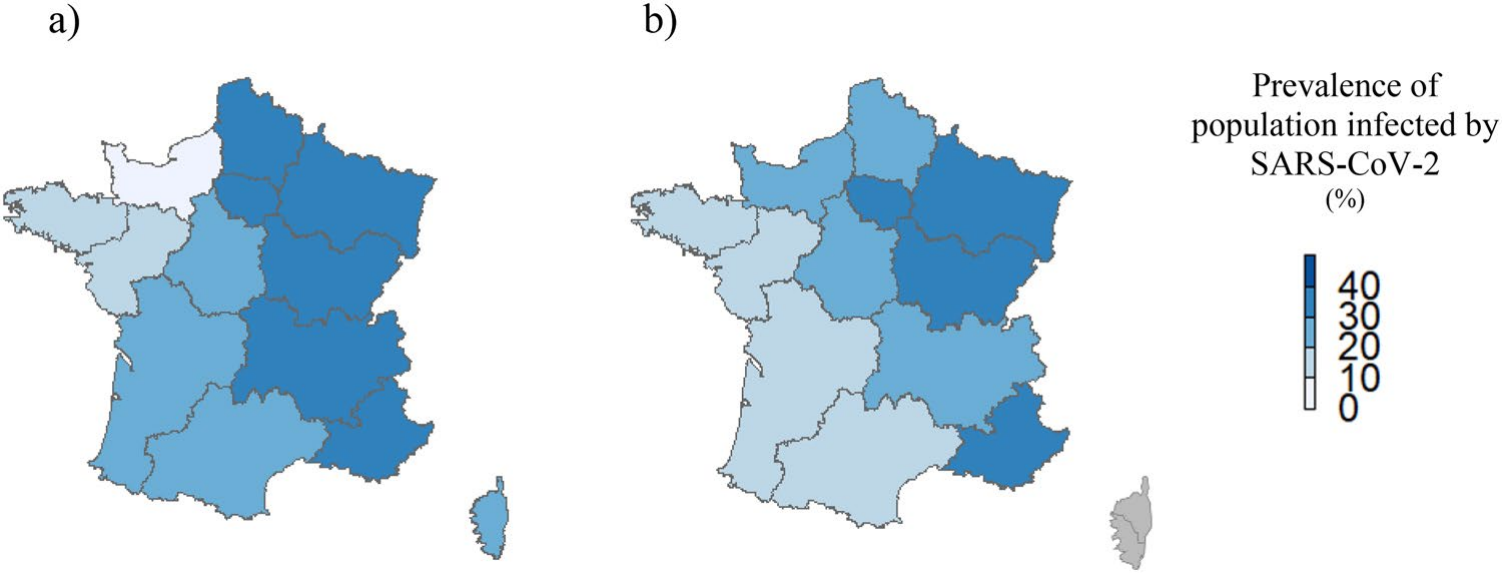
Estimates of (**a**) PHCWs (COVID-SéroPRIM study, France, May–August 2021) and of (**b**) the general adult population (from mathematical modeling) that have been infected by the SARS-CoV-2 in metropolitan France by administrative region after the third COVID-19 wave (as of June 1, 2021). The maps were generated with R software, version 4.0.3 (4.0.3, R Core Team, 2021, R Foundation for Statistical Computing, Vienna, Austria; https://www.r-project.org/).

Estimates of PHCWs and of the general adult population that have been infected by the SARS-CoV-2 at national level are presented in Fig. 2. We found no significant difference between the two (estimates for the general population lied in the confidence intervals of the estimates for PHCWs). Figure 3a,b present the estimations at regional levels (see Supplementary Table S8 for details). There was a strong positive correlation (coefficient of 0.76) between the geographical distribution of the estimates of PHCWs and of the model-based estimates of the general adult population that have been infected by the SARS-CoV-2 (p = 0.004).

Among general practitioners, pediatricians, dentists and pharmacists, region-age weighted estimates are presented with 95% confidence interval, and for modeling from the general adult population with 95% probability range.

Estimates and their 95% confidence intervals or 95% probability range are presented in Supplementary Table S8. Modeling estimates for the regions in gray (Corsica) were not available.

### Factors associated with SARS-CoV-2 infection

Bivariate analyses are presented on Table 2 and showed that there were significant differences in prevalence of SARS-CoV-2 infection according to two non-occupational factors: the region of work place (higher level in Île-de-France [PR = 1.57; CI 95% 1.19–2.09] South-East [aPR = 1.58; CI 95% 1.22–2.09], North-East [PR = 1.72; CI 95% 1.32–2.25]), and among those having an unprotected COVID-19 case contact within the household (PR = 1.36; CI 95% 1.16–1.61). The following occupational factors were also significantly associated with the SARS-CoV-2 infection: PHCWs who were not working in primary care exclusively had higher level of infection than those who worked exclusively in primary care (PR = 1.31; CI 95% 1.08–1.58) and not wearing FFP2 or surgical mask every day during the first lockdown was associated with a higher level of infection compared to those who wore them every day (PR = 1.37; CI 95% 1.02–1.84). GPs had higher prevalence than pharmacist assistants (PR = 2.31; CI 95% 1.01–5.26). Other factors (demographic, clinical, household characteristics, and contact with COVID-19 patient) were not associated with SARS-CoV-2 infection. There were no significant interaction terms.

**Table 2:** Demographic, clinical, household and occupational characteristics of PHCWs and their association with SARS-CoV-2 infection, COVID-SéroPRIM study, France, May–August 2021.

	SARS-CoV-2 infection/total (%)	Unadjusted prevalence ratio	95% CI	*P-*value
Demographic, clinical and household factors				
Age group (years)				
< 40	118/430 (24.4)	Reference		
40–49	132/460 (28.7)	1.05	0.85–1.29	0.6776
50–59	120/420 (28.6)	1.04	0.84–1.29	0.7139
> 60	87/302 (28.8)	1.05	0.83–1.33	0.6849
Sex				
Male	136/500 (27.2)	Reference		
Female	321/1112 (28.9)	1.06	0.89–1.26	0.4942
Chronic diseases				
No	363/1303 (27.9)	Reference		
Yes	94/309 (30.4)	1.09	0.90–1.32	0.3639
Chronic diseases (Yes *vs* No)				
Obesity*	30/106 (28.3)	1.00	0.73–1.37	0.9910
Hypertension	34/117 (29.1)	1.03	0.77–1.38	0.8589
Diabetes	8/21 (38.1)	1.35	0.78–2.34	0.2857
Smoking status				
Smoker	29/138 (21.0)	Reference		
Non-smoker	428/1474 (29.0)	1.38	0.99–1.93	0.0571
Household size and composition				
Nb adults (inc.participant)				
1	44/185 (23.8)	Reference		
2	303/1039 (29.2)	1.23	0.93–1.61	0.1459
3+	110/388 (28.4)	1.19	0.88–1.61	0.2553
Nb children (< 18)				
0	205/724 (28.3)	Reference		
1+	251/885 (28.4)	1.00	0.86–1.17	0.9835
Nb rooms				
1–2	20/84 (23.8)	Reference		
3–4	116/398 (29.1)	1.22	0.81–1.85	0.3361
≥ 5	321/1130(28.4)	1.19	0.80–1.77	0.3793
Community exposure to SARS-CoV-2				
Region*				
North-West	57/304 (18.8)	Reference		
South-West	63/262 (24.0)	1.22	0.90–1.65	0.1251
Île-de-France	91/297 (30.6)	1.57	1.19–2.09	**0.0009**
South-East	112/362 (30.3)	1.58	1.22–2.09	**0.0005**
North-East	134/387 (34.6)	1.72	1.33–2.25	** < 0.0001**
Unprotected COVID-19 case contact ^µ^				
No	323/1236 (26.1)	Reference		
Yes	134/376 (35.6)	1.36	1.16–1.61	**0.0002**
Unprotected COVID-19 case contact (Yes *vs* No)				
Within the household	77/178 (40.4)	1.51	1.24–1.84	** < 0.0001**
During leisure activity	21/67 (31.3)	1.11	0.77–1.60	0.5664
Among colleagues	30/101 (29.7)	1.05	0.77–1.43	0.7532
Occupational factors^$^				
Occupation				0.1759
Pharmacist assistant	5/36 (13.9)	Reference		
Dentist assistant	11/50 (22.0)	1.58	0.60–4.16	0.3509
Pharmacist	59/238 (24.8)	1.78	0.77–4.15	0.1780
Pediatrician	120/430 (27.9)	2.01	0.88–4.60	0.0984
Dentist	93/331 (28.1)	2.02	0.88–4.65	0.0967
General practitioner	169/527 (32.1)	2.31	1.01–5.26	**0.0462**
Work in primary care exclusively				
Yes	373/1375 (27.1)	Reference		
No	84/237 (35.4)	1.31	1.08–1.58	**0.0065**
Number of days worked/week				
< 3	27/111 (24.3)	Reference		
3–4	277/858 (28.9)	1.19	0.84–1.67	0.3230
≥ 5	153/543 (28.2)	1.16	0.81–1.65	0.4164
Care of COVID-19 patients				
No	119/463 (25.7)	Reference		
Yes	338/1149 (29.4)	1.14	0.96–1.37	0.1391
Performance of COVID-19 test				
No	309/1107 (27.9)	Reference		
Yes	138/483 (28.6)	1.02	0.86–1.21	0.2689
Missing	22			
Occupational factors during the first lockdown^$^				
Place of work				
Primary care (exclusively)	288/1038 (27.7)	Reference		
Did not work/remote work	97/324 (29.9)	1.08	0.89–1.31	0.4407
Hospital/COVID-19 center	58/194 (29.9)	1.08	0.85–1.37	0.5365
Missing	56			
Working conditions				
Face-to-face (exclusively)	138/500 (27.6)	Reference		
Remote and face-to-face	222/788 (29.9)	1.02	0.85–1.22	0.8236
Did not work/remote work	97/324 (28.2)	1.08	0.87–1.35	0.4665
Care of COVID-19 patients				
Do not know	64/251 (25.5)	Reference		
No	149/542 (27.5)	1.08	0.84–1.39	0.5581
Yes	244/819 (29.8)	1.17	0.92–1.48	0.1964
Performance of COVID-19 test				
No	393/1392 (28.2)	Reference		
Yes	60/210 (28.6)	1.01	0.80–1.27	0.9189
Missing	10			
PPE use almost every day				
FFP2 or surgical mask				
Yes	330/1203 (28.4)	Reference		
Did not work/remote work	97/324 (29.9)	1.09	0.90–1.32	0.3676
No	30/80 (37.5)	1.37	1.02–1.84	**0.0393**
Missing	5			
Gloves and coat				
Yes	237/867 (27.3)	Reference		
No	122/411 (29.7)	1.09	0.90–1.31	0.3699
Did not work/remote work	97/324 (29.9)	1.10	0.90–1.34	0.3805
Missing	10			
Glasses and coverall				
Yes	64/262 (24.4)	Reference		
No	288/994 (29.0)	1.19	0.94–1.50	0.1531
Did not work/remote work	102/324 (29.9)	1.23	0.94–1.61	0.1403
Missing	32			

Significant values are in bold.

*Body mass index ≥ 30 kg/m^2^.

^µ^Defined as face-to-face contact with a confirmed COVID-19 case, without the use of recommended personal protective equipment.

^$^No occupational-effect modification between SARS-CoV-2 infection and occupational-related factors were found (*p*-value of Wald chi-square test for interaction terms > 0.05).

**Figure 4:**
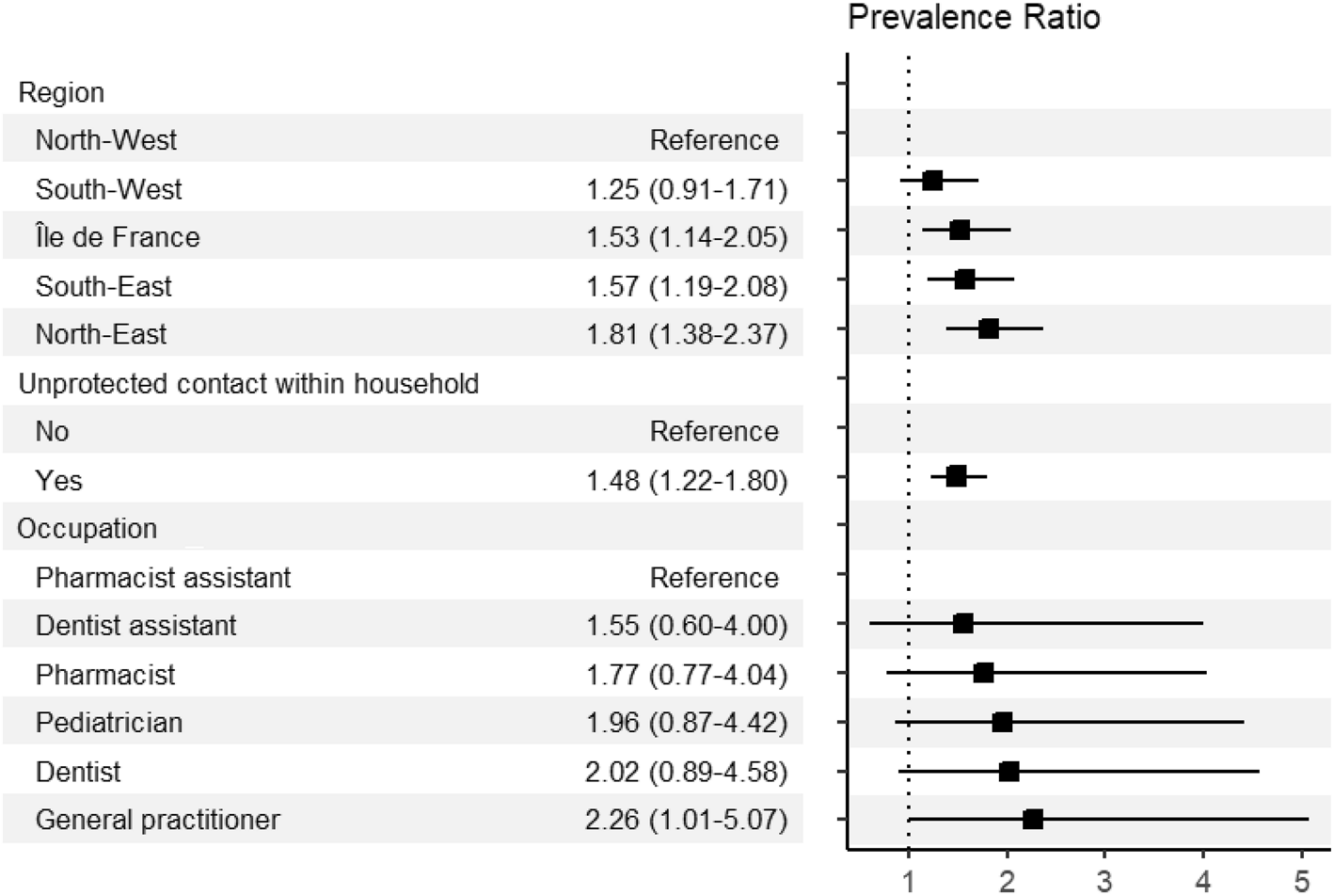
Results of the multivariable analysis of factors associated with SARS-CoV-2 infection among PHCWs*,* COVID-SéroPRIM study, France, May–August 2021. Estimates are prevalence ratio (black square) with 95% confidence intervals.

In the adjusted model, an independent association was found between SARS-CoV-2 infection and region of workplace, unprotected COVID-19 case contact within household, and one occupational group. Compared to PHCWs working in North-West, those working in Île-de-France (adjusted prevalence ratio [aPR] = 1.53; CI 95% 1.14–2.05), South-East (aPR = 1.57; CI 95% 1.19–2.08), and North-East (aPR = 1.81; CI 95% 1.38–2.37) had higher prevalences. PHCWs who had an unprotected COVID-19 case contact had higher prevalence than those who did not (aPR = 1.48; CI 95% 1.22–1.80), and GPs had higher prevalence than pharmacist assistants (aPR = 2.26; CI 95% 1.01–5.07). Multivariate analysis is showed in Fig. 4.

## Discussion

In this study, more than a quarter of French PHCWs have been infected with the SARS-CoV-2 after the third COVID-19 wave. However, we did not observe a higher infection rate among PHCWs compared to the general population. While two non-occupational factors, namely having an unprotected contact with a COVID-19 case within household and working in certain regions of France, were associated with SARS-CoV-2 infection, occupational exposure factors did not show any significant association.

Our estimate of a 28.3% infection for SARS-CoV-2 aligns with the prevalence of 25.4% reported among PHCWs in Belgium during a similar stage of the epidemic^26^. Lower prevalences ranged from 6.2 to 16.5% were reported among GPs in Belgium, in Italy, in community pharmacists in Italy and in Lebanon, and also by a meta-analysis on dental workers infection to SARS-CoV-2^26–28^. These variations were expected, since these studies were conducted at an earlier stage of the pandemic. Our prevalence is lower compared to that reported among physicians and nurses in Kosovo at the end of 2020 (48.63%), likely due to extensive and thorough PCR testing of HCWs in that place or different sampling population^29^. Du to different methodologies, and particularly different definitions of the outcome, the comparison between studies remains limited. Regarding our outcome, we found that 38.3% of PHCWs have been infected with SARS-CoV-2, including 16.6% who were negative to anti-N antibodies but classified as having a history of infection according to the other predefined criteria. This result was expected since anti-N antibodies have been shown to wane quickly in the first months after infection^24^. Most PHCWs who had discordant results between positive serology and self-reported SARS-CoV-2 infection had a positive serology without any known history of infection. This result was also expected given a high proportion of asymptomatic SARS-CoV-2 infection^30^. For those who had a negative serology despite a known history of infection, the discordance of results may be due to a decrease in antibodies that have become undetectable, or to a lack of sensitivity of the serology. Overall, these results highlight the importance of defining the outcome based on multiple to avoid misclassification in SARS-CoV-2 infection history.

Our results suggest that PHCWs were not at higher risk of SARS-CoV-2 infection than the general adult population (19–64 years old) over the first eighteen months of the pandemic. These findings are consistent with some studies^4,6,7^ but conflicting with others^5^. The discrepancies in results may be explained by the study periods. Many studies which reported higher risk of infection among HCWs were conducted during the first wave of the pandemic^3–5^ when frontline healthcare workers faced shortages of PPE in many places and no vaccine available, while most people from the general population were under social restrictions and teleworking. Discordances in results may also been attributed to variations in job-related exposure, access to PPE, community prevalence and implementation of control strategies. Interestingly, we found a lower prevalence of infection-induced antibodies among PHCWs after the third wave of COVID-19 compared to a socially deprived neighborhood in the South of France after the first wave (35.9%)^31^. Together, these results may reflect that PPE use, vaccination, and workplace-based protective measures in care settings can effectively prevent infection.

We found that two non-occupational factors (region of workplace and contact with a COVID-19 case at home) were associated with SARS-CoV-2 infection, while workplace factors did not show any significant association. This adds to the body of evidence that community exposures may be major drivers for infection among HCWs^10^. Not surprisingly, PHCWs living in the most COVID-19-affected area were those having higher prevalences. Since occupational health measures have been implemented consistently across the country, the variability in the prevalence of infected PHCWs according to the regions may be attributed to the COVID-19 community levels (as suggesting by the strong correlation we found between the estimates among the PHCWs and the general population). This is in line with several previous studies^10,13^. Many authors reported a strong positive association between SARS-CoV-2 infection and being in contact with a COVID-19 case at home, including in Switzerland (aOR, 7.79; 95% CI, 5.98–10.15)^9^, in France (aOR 2.00; 95% CI 2.23–3.28) and in Belgium (aOR 3.15; 95% CI 2.33–4.25)^7,14^. In contrast to some studies conducted among hospital HCWs^7^, but consistent with others^10,11^, we did not find associations between the SARS-CoV-2 infection and workplace factors (i.e. number of days worked, care of patients, test performance and PPE use during the first lockdown) in multivariate analysis, albeit two of them were significant in bivariate analyses (higher levels of infection for PHCWs not working exclusively in primary care and for those who were not wearing FFP2 or surgical mask every day during the first lockdown). In France, previous studies reported that close or prolonged contact with patients, aerosol-generating procedures, and performance of upper respiratory tract samples were associated with SARS-CoV-2 infection^7^. Discordance in results might be explained by different study populations, as those studies were conducted among HCWs from hospitals (some served as a referral center for COVID-19 during the first wave). Our result of higher level of SARS-CoV-2 infection among PHCWs who were not working exclusively in primary care are in line with this hypothesis. The lack of power due to a small sample size may explain that the results did not remain significant in multivariate analysis, but it is also possible that the association in bivariable analyses was biased by confounding factors. The same two hypotheses (true association in bivariate analyses but lack of power to show it in multivariate analyses, or biased association in bivariate analyses by confounding factor) may explain the significant association in bivariate analyses between the use of mask and the infection. It was reported in some studies with both occupational and non-occupational factors associated with SARS-CoV-2 infection, that the strongest predictor of contracting COVID-19 was exposure to an infected person outside work^9,14^. Our findings are in lines with the fact that for PHCWs, the risk of SARS-CoV-2 infection from community exposure may be more significant than the risk of occupational exposure.

This study has several strengths. The strengths include national recruitment of PHCWs from different occupations with 85.3% of participants working exclusively in primary care settings, the use of several assays including three serological methods (minimize a potential misclassification bias or underestimation of estimates), and the control for several covariates. This study also has several limitations. Firstly, convenience sampling is a potential source of sampling bias and limit the representativeness of our sample. While we were able to adjust estimates for age and region for GPs, pediatricians, dentists and pharmacists, data were not available for pharmacist and dental assistants in France. In addition, we cannot assure that PHCWs from the networks were representative of the French PHCWs in terms of practices. Furthermore, it is likely that PHCWs who were volunteers were influenced by their history of SARS-CoV-2 infection, which could result in either an underestimate or an overestimate of the true prevalence. Secondly, the small sample sizes may have led to low power to detect differences in estimates, and limit interpretation of the results for dental and pharmacist assistants. Third, PHCWs may have been misclassified with regards to SARS-CoV-2 infection due to the imperfect nature of serological and also because antibody level may wane with time. We might underestimate the actual proportion of infection. Although we included self-reporting history of positive ELISA or PCR to overcome this limitation, undetected infected PHCWs may still be misclassified. Fourth, we recognize that our comparison of estimates between PHCWs and the general population should be taken with caution as they were estimated with different methods. Moreover, modeling study relies on the assumption that infection-hospitalization ratio remains constant over time, which was not the case during the course of the pandemic. However, to our knowledge, there is not statewide data collected during the same period to compare the prevalence of the SARS-CoV-2 infection among PHCWs with that in the general population. Fifth, we cannot exclude recall bias since information was self-reported, including information about practices during the first lockdown. Sixth, we used workplace region as a proxy measure for community exposure, that is area of residence, which may not be accurate due to inter-region travel from home to work. Seventh, residual confounding may exist due to unmeasured factors. Finally, we cannot extrapolate our results to SARS-CoV-2 variant of concerns because our study was conducted prior to the emergence of several variants, including the highly transmissible Delta variant.

This study suggests that PHCWs have not been more infected by SARS-CoV-2 than the general population in metropolitan France after 18 months of pandemic. Factors the most associated with SARS-CoV-2 infection among French PHCWs were non-occupational factors. During the COVID-19 pandemic, PHCWs have had priority access to personal protective equipment at their workplaces. These results hold significant importance for reflecting on policy regarding prevention and control measures. While measures implemented in primary care settings in France seems to be effective at limiting the spread of SARS-CoV-2, the use of adequate protective equipment at home should be further encouraged around a detected case.

### Supplementary Information

Supplementary Information.

## Data Availability

The datasets used and/or analyzed during the current study are available from the corresponding author on reasonable request.

## Abbreviations

aPR: Adjusted prevalence ratio
COVID-19: Coronavirus disease 2019
DBS: Dried-blood collection card
GPs: General practitioners
HCWs: Healthcare workers
N: Nucleocapsid
PHCWs: Primary Healthcare Workers
PPE: Personal protective equipment
ROC: Receiver operating characteristic
RT-qPCR: Reverse transcription-quantitative polymerase chain reaction
S: Spike
VNT: Virus neutralization test
