# Supplementary material for: SARS-CoV-2 infection prevalence and associated factors among primary healthcare workers in France after the third COVID-19 wave
Source: Sci Rep. 2024 Mar 5;14:5418. doi: 10.1038/s41598-024-55477-9 (PMC10914718; doi:10.1038/s41598-024-55477-9)
Supplement: Supplementary file 1 — Supplementary Information. [file 41598_2024_55477_MOESM1_ESM.docx]

**Supplementary Materials**

**Contents:**

[S1 Table. Relative contribution of serology (N-immunoassay, ELISA-S, SN assay) on DBS samples and self-reported infection (PCR, ELISA) to overall number of infections among primary healthcare workers (N=1612), COVID-19-SéroPRIM study, France, 2021. 2](#_Toc139635692)

[S2 Table. Age- and region- distribution of general practitioners, pediatricians and dentists in primary care, and community pharmacists in France (respectively: N= 59159, N= 2089, N=34690, N= 27875), in SéroPRIM sample (respectively: n= 527, n= 430, n= 331, n= 238), and weights per individual for each age-region stratum. 3](#_Toc139635693)

[S3 Table. Demographic, clinical, household and occupational characteristics of primary health care workers by occupational group (N=1612), COVID-19-SéroPRIM study, France, 2021. 4](#_Toc139635694)

[S4 Table. Self-reported SARS-CoV-2 infection (positive PCR or ELISA) according to serology results on collected DBS sample (positive N-immunoassay, or, in vaccinated individuals, positive ELISA-S or SN assay) 7](#_Toc139635695)

[S5 Table. Self-reported SARS-CoV-2 infection (positive PCR or ELISA) and serology results on collected DBS sample (positive N-immunoassay, or, in vaccinated individuals, positive ELISA-S or SN assay) according to history of COVID-19 vaccine. 7](#_Toc139635696)

[S6 Figure. Number of positive RT-qPCR retrospectively self-reported by PHCWs during the COVID-SéroPRIM study in conjunction with the number of new daily confirmed COVID-19 hospital admissions (data from French government/www.data.gouv.fr). 7](#_Toc139635697)

[S7 Table. SARS-CoV-2 infection among primary healthcare workers by occupation (N=1612), COVID-19-SéroPRIM study, France, 2021. 8](#_Toc139635698)

[S8 Table. Estimates of primary healthcare workers (COVID-SéroPRIM study, France, May–August 2021) and of the general adult population (from modeling) that have been infected by the SARS-CoV-2 by administrative region in metropolitan France after the third COVID-19 wave (May-August 2021). 8](#_Toc139635699)

# S1 Table. Relative contribution of serology (N-immunoassay, ELISA-S, SN assay) on DBS samples and self-reported infection (PCR, ELISA) to overall number of infections among primary healthcare workers (N=1612), COVID-19-SéroPRIM study, France, 2021.

|  | Number (proportion)  N=1612 | Proportion among total infections (n=457) |
| --- | --- | --- |
| Serology on DBS samples |  |  |
| Anti-N Abs positive | 381 (23.6) | 83.4 |
| Anti-S or SN Abs positive in unvaccinated individuals (negative for anti-N Abs) | 13 (0.8) | 2.8 |
| Self-reporting infection |  |  |
| PCR/antigenic positive (negative for serologies on DBS sample) | 45 (2.8) | 9.9 |
| ELISA positive (negative or not tested for PCR/antigenic; before vaccination) | 18 (1.1) | 3.9 |
| Total infections | 457 (28.3%) |  |

N-immunoassay: immunoassays detecting antibodies against SARS-CoV-2 nucleocapsid; ELISA-S: ELISA test detecting antibodies against the S-1 portion of the S protein of the SARS-CoV-2: DBS: dried blood spot; PCR – polymerase chain reaction; Abs: antibodies; anti-N: anti-Nucleocapsid; anti-S: anti-Spike; SN: seroneutralization.

# S2 Table. Age- and region- distribution of general practitioners, pediatricians and dentists in primary care, and community pharmacists in France (respectively: N= 59159, N= 2089, N=34690, N= 27875), in SéroPRIM sample (respectively: n= 527, n= 430, n= 331, n= 238), and weights per individual for each age-region stratum.

Each stratum size for the French PHCWs refer to the estimated population from the French’s Research, Study, Evaluation and Statistics Direction (Dress <https://drees.shinyapps.io/demographie-ps/>)

|  | French population | | | |  | SéroPRIM sampling | | | |  | Weights | | | |
| --- | --- | --- | --- | --- | --- | --- | --- | --- | --- | --- | --- | --- | --- | --- |
|  | Age | | | |  | Age | | | |  | Age | | | |
|  | < 40 | 40-49 | 50-59 | ≥60 |  | < 40 | 40-49 | 50-59 | ≥60 |  | < 40 | 40-49 | 50-59 | ≥60 |
| General practitioners | |  |  |  |  |  |  |  |  |  |  |  |  |  |
| Île de France | 2057 | 1397 | 2244 | 4034 |  | 28 | 9 | 17 | 15 |  | 0.654 | 1.383 | 1.176 | 2.396 |
| North West | 3879 | 2082 | 2753 | 3682 |  | 28 | 24 | 32 | 11 |  | 1.234 | 0.773 | 0.766 | 2.982 |
| North East | 3679 | 2152 | 3448 | 4463 |  | 42 | 31 | 46 | 33 |  | 0.780 | 0.618 | 0.668 | 1.205 |
| South East | 4135 | 2490 | 3289 | 4771 |  | 40 | 33 | 26 | 26 |  | 0.921 | 0.672 | 1.127 | 1.635 |
| South West | 1792 | 1662 | 2745 | 2405 |  | 30 | 24 | 18 | 14 |  | 0.532 | 0.617 | 1.358 | 1.530 |
| Pediatricians |  |  |  |  |  |  |  |  |  |  |  |  |  |  |
| Île de France | 146 | 160 | 202 | 400 |  | 10 | 19 | 25 | 44 |  | 2.032 | 1.172 | 1.125 | 1.265 |
| North West | 96 | 110 | 95 | 111 |  | 20 | 23 | 10 | 14 |  | 0.668 | 0.666 | 1.322 | 1.104 |
| North East | 89 | 122 | 99 | 181 |  | 11 | 25 | 21 | 27 |  | 1.126 | 0.679 | 0.656 | 0.933 |
| South East | 176 | 153 | 160 | 238 |  | 16 | 29 | 26 | 25 |  | 1.531 | 0.734 | 0.857 | 1.325 |
| South West | 163 | 120 | 104 | 164 |  | 17 | 24 | 18 | 26 |  | 1.335 | 0.696 | 0.804 | 0.878 |
| Dentists |  |  |  |  |  |  |  |  |  |  |  |  |  |  |
| Île de France | 3121 | 1741 | 2110 | 1656 |  | 22 | 25 | 27 | 12 |  | 1.354 | 0.664 | 0.746 | 1.317 |
| North West | 1905 | 1096 | 1483 | 1137 |  | 18 | 23 | 9 | 7 |  | 1.010 | 0.455 | 1.572 | 1.550 |
| North East | 1432 | 915 | 1162 | 921 |  | 13 | 10 | 20 | 5 |  | 1.051 | 0.873 | 0.554 | 1.758 |
| South East | 2956 | 1729 | 2051 | 1838 |  | 34 | 19 | 28 | 8 |  | 0.830 | 0.868 | 0.699 | 2.192 |
| South West | 2630 | 1493 | 1776 | 1538 |  | 13 | 22 | 11 | 5 |  | 1.930 | 0.648 | 1.541 | 2.935 |
| Pharmacists |  |  |  |  |  |  |  |  |  |  |  |  |  |  |
| Île de France | 964 | 1072 | 1176 | 1227 |  | 7 | 14 | 11 | 7 |  | 1.354 | 0.664 | 0.746 | 1.317 |
| North West | 1056 | 1491 | 1607 | 1124 |  | 19 | 22 | 17 | 7 |  | 1.010 | 0.455 | 1.572 | 1.550 |
| North East | 1266 | 1745 | 1676 | 1282 |  | 22 | 30 | 17 | 8 |  | 1.051 | 0.873 | 0.554 | 1.758 |
| South East | 1299 | 1614 | 1868 | 1548 |  | 8 | 6 | 12 | 6 |  | 1.386 | 2.297 | 1.329 | 2.203 |
| South West | 1135 | 1629 | 1737 | 1359 |  | 4 | 14 | 6 | 1 |  | 2.423 | 0.993 | 2.472 | 11.603 |

# S3 Table. Demographic, clinical, household and occupational characteristics of primary health care workers by occupational group (N=1612), COVID-19-SéroPRIM study, France, 2021.

|  | | | Total  N= 1612 | GPs  n= 527 | Pediatricians  n= 430 | Dentists  n= 331 | Dental assistants  n= 50 | Pharmacists  n= 238 | Pharmacist assistants n=36 | *P-*value |
| --- | --- | --- | --- | --- | --- | --- | --- | --- | --- | --- |
|  | | | n (%) | n (%) | n (%) | n (%) | n (%) | n (%) | n (%) |  |
|  |  | Demographic, clinical and household factors | | | | | | | | |
| Age group (years) | | |  |  |  |  |  |  |  | <0.0001 |
| <40 | | | 430 (26.7) | 168 (31.9) | 74 (17.2) | 100 (30.2) | 17 (34.0) | 60 (25.2) | 11 (30.6) |  |
| 40-49 | | | 460 (28.5) | 121 (23.0) | 120 (27.9) | 99 (29.9) | 23 (46.0) | 86 (36.1) | 11 (30.6) |  |
| 50-59 | | | 420 (26.1) | 139 (26.4) | 100 (23.3) | 95 (28.7) | 10 (20.0) | 63 (26.5) | 13 (36.1) |  |
| >60 | | | 302 (18.7) | 99 (18.8) | 136 (31.6) | 37(11.2) | 0 (0) | 29 (12.2) | 1 (2.8) |  |
| Gender | | |  |  |  |  |  |  |  | <0.0001 |
| Female | | | 1112 (69.0) | 291 (55.2) | 359 (83.5) | 221 (66.8) | 49 (98.0) | 161 (67.6) | 31 (86.1) |  |
| Male | | | 500 (31.0) | 236 (44.8) | 71 (16.5) | 110 (33.2) | 1 (2.0) | 77 (32.4) | 5 (13.9) |  |
| Chronic diseases | | |  |  |  |  |  |  |  | 0.0016 |
| No | | | 1303 (80.8) | 408 (77.4) | 335 (77.9) | 280 (84.6) | 45 (90.0) | 208 (87.4) | 27 (75.0) |  |
| Yes | | | 309 (19.2) | 119 (22.6) | 95 (22.1) | 51 (15.4) | 5 (10.0) | 30 (12.6) | 9 (25.0) |  |
| Chronic diseases (Yes *vs* No) | | |  |  |  |  |  |  |  |  |
| Obesity* | | | 106 (6.6) | 48 (9.1) | 24 (5.6) | 17 (5.1) | 1 (2.0) | 12 (5.0) | 4 (11.1) | 0.0475 |
| Hypertension | | | 117 (7.3) | 47 (38.3) | 38 (8.8) | 15 (4.5) | 1 (2.0) | 12 (5.0) | 4 (11.1) | 0.0235 |
| Diabetes | | | 21 (1.3) | 6 (1.1) | 6 (5.6) | 5 (1.5) | 0 (0) | 3 (1.3) | 1 (2.8) | 0.8326 |
| Smoking status | | |  |  |  |  |  |  |  | 0.0005 |
| Non-smoker | | | 1474 (91.4) | 493 (93.5) | 404 (94.0) | 288 (87.0) | 40 (80.0) | 218 (91.6) | 31 (86.1) |  |
| Smoker | | | 138 (8.6) | 34 (6.5) | 26 (6.0) | 43 (13.0) | 10 (20.0) | 20 (8.4) | 5 (13.9) |  |
| Household size and composition | | |  |  |  |  |  |  |  |  |
| Nb adults (inc. participant) | | |  |  |  |  |  |  |  | 0.5302 |
| 1 | | | 185 (11.5) | 52 (9.9) | 49 (11.4) | 50 (15.1) | 6 (12.0) | 25 (10.5) | 3 (8.3) |  |
| 2 | | | 1039 (64.5) | 356 (67.6) | 268 (62.3) | 206 (62.2) | 30 (60.0) | 155 (65.1) | 24 (66.7) |  |
| 3+ | | | 388 (24.1) | 119 (22.6) | 113 (26.3) | 75 (22.7) | 14 (28.0) | 58 (24.4) | 9 (25.0) |  |
| Nb children (<18) | | |  |  |  |  |  |  |  | 0.2083 |
| 0 | | | 724 (55.2) | 229 (53.3) | 200 (55.7) | 159 (48.0) | 15 (30.0) | 103 (43.6) | 18 (50.0) |  |
| 1+ | | | 588 (44.8) | 98 (44.7) | 159 (44.3) | 172 (52.0) | 35 (70.0) | 133 (56.4) | 18 (50.0) |  |
| Missing | | | 3 |  |  |  |  |  |  |  |
| Nb rooms | | | | |  |  |  |  |  | 0.0005 |
| 1-2 | | | 84 (5.2) | 75 (14.2) | 40 (9.3) | 29 (8.8) | 2 (4.0) | 11 (4.6) | 3 (8.3) |  |
| 3 | | | 161 (10.0) | 295 (56.0) | 265 (61.6) | 98 (29.6) | 27 (54.0) | 59 (24.8) | 16 (44.4) |  |
| ≥4 | | | 1367 (84.8) | 157 (29.8) | 125 (29.1) | 204 (61.6) | 21 (42.0) | 168 (70.6) | 17 (47.2) |  |

S3 Table. Continued.

|  | Total  N= 1612 | GPs  n= 527 | Pediatricians  n= 430 | Dentists  n= 331 | Dental assistants  n= 50 | Pharmacists  n= 238 | Pharmacist assistants n=36 | *P-*value |
| --- | --- | --- | --- | --- | --- | --- | --- | --- |
|  | n (%) | n (%) | n (%) | n (%) | n (%) | n (%) | n (%) |  |
| Community exposure to SARS-CoV-2 | | | | | | | | |
| Region |  |  |  |  |  |  |  | <0.0001 |
| Île-de-France | 297 (18.4) | 95 (18.0) | 67 (15.6) | 86 (26.0) | 4 (8.0) | 39 (16.4) | 1 (2.8) |  |
| North-West | 304 (18.9) | 69 (13.1) | 98 (22.8) | 57 (17.2) | 8 (16.0) | 65 (27.3) | 12 (33.3) |  |
| North -East | 387 (24.0) | 152 (28.8) | 84 (19.5) | 48 (14.5) | 11 (22.0) | 77 (32.4) | 15 (41.7) |  |
| South-East | 362 (22.5) | 125 (23.7) | 96 (22.3) | 89 (26.9) | 16 (32.0) | 32 (13.4) | 4 (11.1) |  |
| South-West | 262 (16.3) | 86 (16.3) | 85 (19.8) | 51 (15.4) | 11 (22.0) | 25 (10.5) | 4 (11.1) |  |
| Unprotected contact with a COVID-19 case^µ^ | |  |  |  |  |  |  | 0.3192 |
| No | 1236 (76.7) | 399 (75.7) | 325 (75.6) | 249 (75.2) | 38 (76.0) | 194 (81.5) | 31 (86.1) |  |
| Yes | 376 (23.3) | 128 (24.3) | 105 (24.4) | 82 (24.8) | 12 (24.0) | 44 (18.5) | 5 (13.9) |  |
| Unprotected contact with a COVID-19 case (Yes *vs* No) | | |  |  |  |  |  |  |
| Within household | 178 (11.0) | 51 (9.7) | 59 (13.7) | 298 (90.0) | 44 (88.0) | 213 (89.5) | 32 (88.0) | 0.0400 |
| Among colleagues | 101 (6.3) | 36 (6.8) | 25 (5.8) | 26 (7.9) | 3 (6.0) | 11 (4.6) | 0 (0) | 0.4093 |
| During leisure activity | 67 (4.2) | 22 (4.2) | 14 (3.3) | 21 (6.3) | 1 (2.0) | 9 (3.8) | 0 (0) | 0.2929 |
| During meal | 50 (3.1) | 17 (3.2) | 12 (2.8) | 12 (3.6) | 1 (2.0) | 8 (3.4) | 0 (0) | 0.9590 |
| Other | 12 (0.7) | 3 (0.6) | 3 (0.7) | 4 (1.2) | 0 (0) | 2 (0.8) | 0 (0) | 0.8921 |
| At sport | 7 (0.4) | 2 (0.4) | 0 (0) | 5 (1.5) | 0 (0) | 0 (0) | 0 (0) | 0.0760 |
| Missing | 56 |  |  |  |  |  |  |  |
| Occupational factors since the first lockdown | | | | | | | | |
| Work in primary care exclusively |  |  |  |  |  |  |  | <0.0001 |
| No | 237 (14.7) | 57 (10.8) | 132 (30.7) | 41 (12.4) | 0 (0) | 7 (12.4) | 0 (0) |  |
| Yes | 1375 (85.3) | 470 (89.2) | 298 (69.3) | 290 (87.6) | 50 (100) | 231 (87.6) | 35 (100) |  |
| Number of days worked/week |  |  |  |  |  |  |  | <0.0001 |
| <3 | 111 (6.9) | 32 (6.1) | 45 (10.5) | 20 (6.0) | 5 (10.0) | 5 (2.1) | 4 (11.1) |  |
| 3-4 | 958 (59.4) | 282 (53.5) | 284 (66.0) | 258 (77.9) | 37 (31.9) | 76 (31.9) | 21 (58.3) |  |
| ≥5 | 543 (33.7) | 213 (40.4) | 101 (23.5) | 53 (16.0) | 8 (66.0) | 157 (66.0) | 11 (30.6) |  |
| Care of COVID-19 patients |  |  |  |  |  |  |  | <0.0001 |
| No | 463 (28.7) | 16 (3.0) | 139 (32.3) | 253 (76.4) | 44 (88.0) | 6 (2.5) | 5 (13.9) |  |
| Yes | 1149 (71.3) | 511 (97.0) | 291 (67.7) | 78 (23.6) | 6 (12.0) | 232 (97.5) | 31 (86.1) |  |
| Performance of COVID-19 test | |  |  |  |  |  |  | <0.0001 |
| No | 1107 (69.6) | 300 (58.3) | 283 (67.1) | 322 (97.3) | 49 (98.0) | 122 (51.5) | 31 (88.6) |  |
| Yes | 483 (30.4) | 215 (41.7) | 139 (32.9) | 9 (2.7) | 1 (2.0) | 115 (48.5) | 4 (11.4) |  |
| Missing | 22 |  |  |  |  |  |  |  |

S3 Table. Continued.

|  | Total  N= 1612 | GPs  n= 527 | Pediatricians  n= 430 | Dentists  n= 331 | Dental assistants  n= 50 | Pharmacists  n= 238 | Pharmacist assistants n=36 | *P-*value |
| --- | --- | --- | --- | --- | --- | --- | --- | --- |
|  | n (%) | n (%) | n (%) | n (%) | n (%) | n (%) | n (%) |  |
|  | Occupational factors during the first lockdown | | | | | | | |
| Place of work |  |  |  |  |  |  |  | 0.0005 |
| Did not work/remote work | 324 (20.8) | 24 (4.7) | 18 (4.4) | 232 (70.5) | 42 (100) | 6 (2.6) | 2 (5.6) |  |
| Primary care (exclusively) | 1038 (66.7) | 412 (81.3) | 300 (73.3) | 66 (20.1) | 0 (0) | 226 (97.0) | 34 (94.4) |  |
| Hospital/COVID-19 center | 194 (12.5) | 71 (14.0) | 91 (22.2) | 31 (9.4) | 0 (0) | 1 (4.3) | 0 (0) |  |
| Missing | 56 |  |  |  |  |  |  | <0.0001 |
| Working conditions |  |  |  |  |  |  |  |  |
| Did not work/remote work | 324 (20.1) | 24 (4.6) | 18 (4.2) | 232 (70.1) | 42 (84.0) | 6 (2.5) | 2 (5.6) |  |
| Remote and face-to-face | 788 (48.9) | 400 (75.9) | 316 (73.5) | 70 (21.1) | 1 (2.0) | 1 (0.4) | 0 (0) |  |
| Face-to-face (exclusively) | 500 (31.0) | 103 (19.5) | 96 (19.5) | 29 (8.8) | 7 (14.0) | 231 (97.1) | 34 (94.4) |  |
| Care of COVID-19 patients |  |  |  |  |  |  |  | <0.0001 |
| No | 542 (33.6) | 55 (10.4) | 160 (37.2) | 263 (79.5) | 46 (92.0) | 13 (5.5) | 5 (13.9) |  |
| Yes | 819 (50.8) | 439 (83.3) | 159 (37.0) | 14 (4.2) | 1 (2.0) | 183 (76.9) | 23 (63.9) |  |
| Don’t know | 251 (15.6) | 33 (6.3) | 111 (25.8) | 54 (16.3) | 3 (6.0) | 52 (17.6) | 8 (22.2) | <0.0001 |
| Performance of COVID-19 test |  |  |  |  |  |  |  |  |
| No | 1392 (86.9) | 406 (77.6) | 368 (85.6) | 326 (98.5) | 50 (100) | 208 (89.3) | 34 (97.1) |  |
| Yes | 210 (13.1) | 117 (68.6) | 62 (14.4) | 5 (1.5) | 0 (0) | 25 (10.7) | 1 (2.9) |  |
| Missing | 10 |  |  |  |  |  |  |  |
| PPE use almost every day |  |  |  |  |  |  |  | 0.0005 |
| FFP2 or surgical mask |  |  |  |  |  |  |  |  |
| Did not work/remote work | 324 (20.2) | 24 (4.6) | 18 (4.2) | 232 (70.7) | 42 (84.0) | 6 (2.5) | 2 (5.6) |  |
| No | 80 (5.0) | 27 (5.1) | 21 (4.9) | 10 (3.0) | 1 (2.0) | 17 (7.1) | 4 (11.1) |  |
| Yes | 1203 (74.9) | 474 (90.3) | 391 (90.0) | 86 (26.2) | 7 (14.0) | 215 (90.3) | 30 (83.3) |  |
| Missing | 5 |  |  |  |  |  |  |  |
| Gloves and coat |  |  |  |  |  |  |  | <0.0001 |
| Did not work/remote work | 324 (20.2) | 24 (4.6) | 18 (4.2) | 232 (70.7) | 42 (84.0) | 6 (2.5) | 2 (5.6) |  |
| No | 411 (25.7) | 133 (25.4) | 158 (36.9) | 18 (5.1) | 1 (2.0) | 95 (40.3) | 7 (21.2) |  |
| Yes | 867 (54.1) | 367 (70.0) | 252 (58.9) | 82 (24.8) | 7 (14.0) | 135 (57.2) | 24 (72.7) |  |
| Missing | 10 |  |  |  |  |  |  |  |
| Glasses and coverall |  |  |  |  |  |  |  | <0.0001 |
| Did not work/remote work | 324 (20.5) | 24 (4.6) | 18 (4.2) | 232 (70.7) | 42 (84.0) | 6 (2.5) | 2 (5.6) |  |
| No | 994 (62.9) | 389 (75.4) | 348 (82.7) | 17 (5.2) | 2 (4.0) | 209 (90.9) | 29 (87.9) |  |
| Yes | 262 (16.6) | 103 (20.0) | 55 (13.1) | 81 (24.5) | 6 (12.0) | 15 (5.5) | 2 (6.1) |  |
| Missing | 32 |  |  |  |  |  |  |  |

*Body mass index ≥ 30 kg/m2. ^µ^Defined as face-to-face contact with a confirmed COVID-19 case, without the use of recommended personal protective equipment.

# S4 Table. Self-reported SARS-CoV-2 infection (positive PCR or ELISA) according to serology results on collected DBS sample (positive N-immunoassay, or, in vaccinated individuals, positive ELISA-S or SN assay)

| Self-reported SARS-CoV-2 infection | Positive serology | |
| --- | --- | --- |
|  | No (n= 1218) | Yes (n= 394) |
| No (n=1359) | 1155 (94.8) | 204 (51.8) |
| Yes (n= 253) | 63 (5.2) | 190 (48.2) |

PCR: polymerase chain reaction; DBS: dried blood spot; N-immunoassay: immunoassays detecting antibodies against SARS-CoV-2 nucleocapsid; ELISA-S: ELISA test detecting antibodies against the S-1 portion of the S protein of the SARS-CoV-2.

# S5 Table. Self-reported SARS-CoV-2 infection (positive PCR or ELISA) and serology results on collected DBS sample (positive N-immunoassay, or, in vaccinated individuals, positive ELISA-S or SN assay) according to history of COVID-19 vaccine.

|  | At least one dose  of COVID-19 vaccine | |
| --- | --- | --- |
|  | No (N=124) | Yes (N=1,488) |
| Positive PCR/antigenic test | 46 (37.1) | 145 (9.7) |
| Positive ELISA test | 35 (28.2) | 88 (5.9) |
| ELISA-S positive | 53 (42.7) | 1,473 (99.0) |
| NP- positive | 59 (47.6) | 322 (21.6) |
| Positive seroneutralization assay | 43 (34.7) | 1,268 (85.2) |
| Total history of SARS-CoV-2 infection | 74 (59.7) | 383 (25.7) |

# S6 Figure. Number of positive RT-qPCR retrospectively self-reported by PHCWs during the COVID-SéroPRIM study in conjunction with the number of new daily confirmed COVID-19 hospital admissions (data from French government[/www.data.gouv.fr](https://www.data.gouv.fr)).


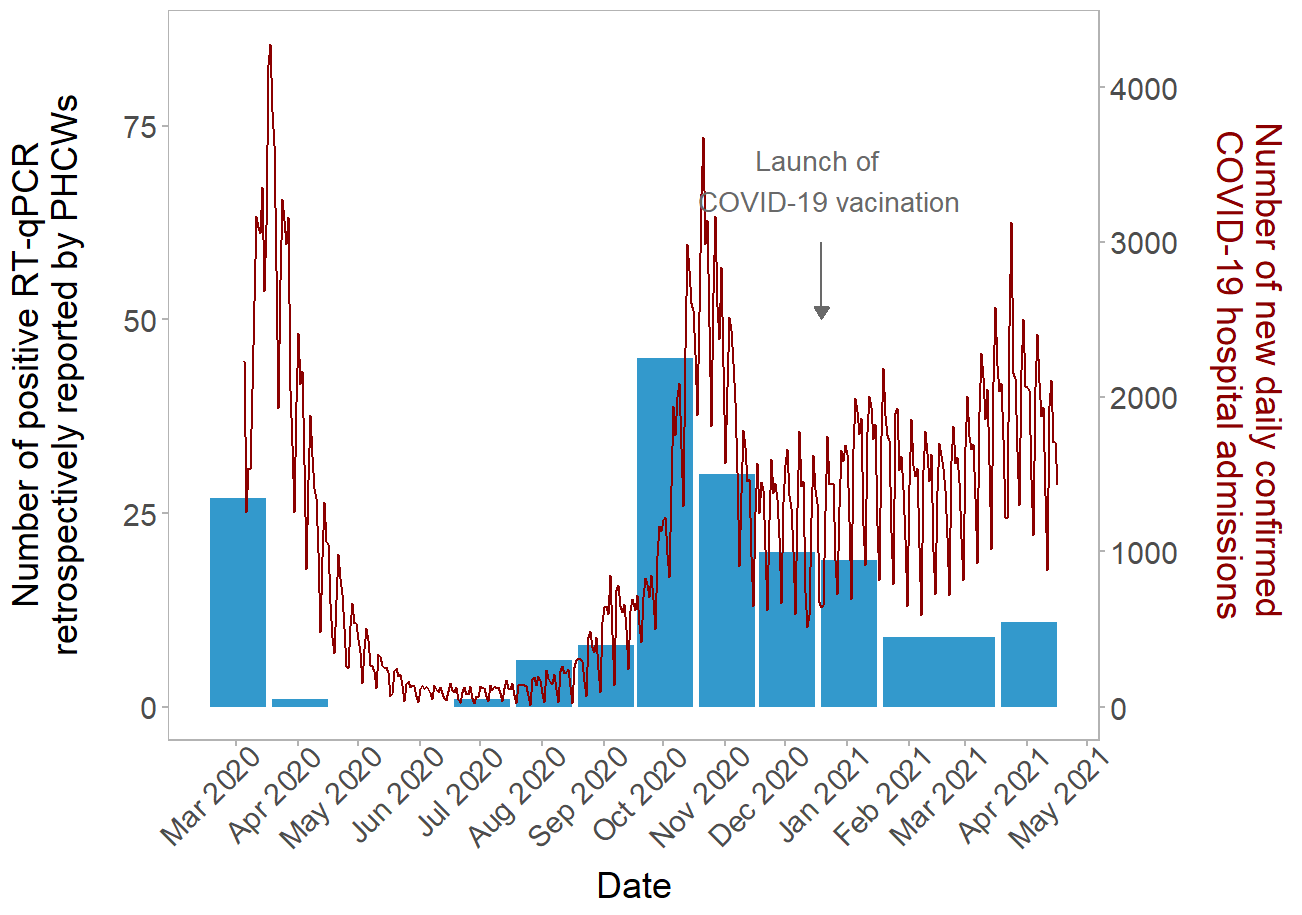


Rationale for this analysis: Since the history of RT-qPCR were self-reported by PHCWs for the previous eighteen months (possible recall bias), we explored the validity of this report by describing the number of reported positive RT-qPCR by month of diagnosis, in conjunction with the number of new daily confirmed COVID-19 hospital admissions (data from French government [/www.data.gouv.fr](https://www.data.gouv.fr)). New daily confirmed COVID-19 hospital admissions were chosen as a proxy of the COVID-19 waves since it is the only available data from the beginning of the pandemic.

# S7 Table. SARS-CoV-2 infection among primary healthcare workers by occupation (N=1612), COVID-19-SéroPRIM study, France, 2021.

|  | Total  N=1612 | GPs  n= 527 | Pediatricians  n= 430 | Dentists  n= 331 | Dental assistants  n= 50 | Pharmacists  n= 238 | Pharmacist assistants  n= 36 | *P-*value |
| --- | --- | --- | --- | --- | --- | --- | --- | --- |
|  | n (%) | n (%) | n (%) | n (%) | n (%) | n (%) | n (%) |  |
| Self-reporting infection | 253 (15.7) | 99 (18.8) | 66 (15.3) | 48 (14.5) | 4 (8.0) | 34 (14.3) | 2 (5.6) | 0.09 |
| Positive PCR/antigenic test | 191 (11.8) | 78 (14.8) | 54 (12.6) | 33 (10.0) | 3 (6.0) | 22 (9.2) | 1 (2.8) | 0.04 |
| Period of positive tests |  |  |  |  |  |  |  |  |
| Mar -Apr 31, 2020 | 28 (1.7) | 13 (2.5) | 5 (1.2) | 7 (2.1) | 0 (0) | 3 (1.3) | 0 (0) | 0.64 |
| May 1^st^ - Aug 31, 2020 | 7 (0.4) | 3 (0.6) | 1 (0.2) | 2 (0.6) | 1 (2.0) | 0 (0) | 0 (0) | 0.38 |
| Sep 1^st^ - Dec 31 2020 | 104 (6.5) | 43 (8.2) | 30 (7.0) | 16 (4.8) | 1 (2.0) | 14 (5.9) | 0 (0) | 0.17 |
| Jan 1^st^ – Apr 30 2021 | 50 (3.1) | 20 (3.8) | 19 (4.4) | 5 (1.5) | 1 (2.0) | 5 (2.1) | 0 (0) | 0.18 |
| Missing | 2 |  |  |  |  |  |  |  |
| Strain (Yes *vs* No) |  |  |  |  |  |  |  |  |
| Historical strain | 99 (51.8) | 45 (8.5) | 25 (5.8) | 17 (5.1) | 2 (4.0) | 9 (3.8) | 1 (2.8) | 0.13 |
| Alpha | 21 (1.3) | 4 (0.8) | 9 (2.1) | 6 (1.6) | 0 (0) | 2 (0.7) | 0 (0) | 0.46 |
| Other | 2 (0.1) | 0 (0) | 1 (0.2) | 1 (0.3) | 0 (0) | 0 (0) | 0 (0) | 0.69 |
| Missing |  |  |  |  |  |  |  |  |
| Positive ELISA test | 123 (7.6) | 47 (8.9) | 30 (7.0) | 27 (8.2) | 1 (2.0) | 17 (7.1) | 1 (2.8) | 0.58 |
| Infection-induced antibodies from DBS analysis | 394 (24.4) | 141 (26.8) | 106 (24.7) | 84 (25.4) | 11 (22.0) | 47 (19.7) | 5 (13.9) | 0.23 |
| N-immunoassay | 381 (23.6) | 138 (26.2) | 104 (24.2) | 77 (23.3) | 11 (22.0) | 46 (19.3) | 5 (13.9) | 0.27 |
| ELISA-S | 53 (3.3) | 17 (3.2) | 10 (2.3) | 16 (4.8) | 2 (4.0) | 7 (2.9) | 1 (2.8) | 0.50 |
| SN assay | 43 (2.7) | 14 (2.7) | 9 (2.1) | 15 (4.5) | 1 (2.0) | 3 (1.3) | 1 (2.8) | 0.23 |

PCR: polymerase chain reaction; DBS: dried blood spot; N-immunoassay: immunoassays detecting antibodies against SARS-CoV-2 nucleocapsid; ELISA-S: ELISA test detecting antibodies against the S-1 portion of the S protein of the SARS-CoV-2; SN: seroneutralization.

# S8 Table. Estimates of primary healthcare workers (COVID-SéroPRIM study, France, May–August 2021) and of the general adult population (from modeling) that have been infected by the SARS-CoV-2 by administrative region in metropolitan France after the third COVID-19 wave (May-August 2021).

| Region |  | PHCWs | | | | General population | | | | |
| --- | --- | --- | --- | --- | --- | --- | --- | --- | --- | --- |
|  | n | % | 95% confidence interval | |  | | % | | 95% probability range | |
| Auvergne-Rhône-Alpes | 267 | 30.3 | (24.8; 35.9) |  |  | | 29.7 | (23.3; | | 35.7) |
| Hauts-de-France | 172 | 36.6 | (29.4;43.8) |  |  | | 29.4 | (21.6; | | 37.0) |
| Provence-Alpes-Côte d'Azur | 91 | 33.0 | (23.3;42.6) |  |  | | 31.4 | (25.1; | | 38.2) |
| Grand Est | 153 | 34.0 | (26.5;41.5) |  |  | | 34.6 | (21.8; | | 49.1) |
| Occitanie | 130 | 21.5 | (14.5; 28.6) |  |  | | 16.9 | (13.5; | | 19.9) |
| Normandie | 57 | 8.8 | (1.4; 16.1) |  |  | | 20.2 | (15.4; | | 24.5) |
| Nouvelle-Aquitaine | 132 | 26.5 | (19.0; 34.0) |  |  | | 14.0 | (11.0; | | 16.6) |
| Centre-Val de Loire | 67 | 28.4 | (17.6;39.2) |  |  | | 22.5 | (16.9; | | 27.5) |
| Bourgogne-Franche-Comté | 62 | 30.6 | (19.2;42.1) |  |  | | 33.2 | (25.4; | | 40.5) |
| Bretagne | 128 | 18.0 | (11.3;24.6) |  |  | | 11.7 | (9.0; | | 14.2) |
| Pays de la Loire | 108 | 13.9 | (7.4;20.4) |  |  | | 16.7 | (12.8; | | 20.4) |
| Île-de-France | 241 | 35.7 | (29.6;41.7) |  |  | | 36.1 | (24.3; | | 48.1) |
